# Are the current gRNA ranking prediction algorithms useful for genome editing in plants?

**DOI:** 10.1371/journal.pone.0227994

**Published:** 2020-01-24

**Authors:** Fatima Naim, Kylie Shand, Satomi Hayashi, Martin O’Brien, James McGree, Alexander A. T. Johnson, Benjamin Dugdale, Peter M. Waterhouse

**Affiliations:** 1 Centre for Tropical Crops and Biocommodities, Science and Engineering Faculty, Queensland University of Technology, Brisbane, Queensland, Australia; 2 Centre for Crop and Disease Management, School of Molecular and Life Sciences, Curtin University, Bentley, Western Australia, Australia; 3 School of BioSciences, The University of Melbourne, Melbourne, Victoria, Australia; 4 School of Mathematical Sciences, Science and Engineering Faculty, Queensland University of Technology, Brisbane, Queensland, Australia; West China Hospital, Sichuan University, CHINA

## Abstract

Introducing a new trait into a crop through conventional breeding commonly takes decades, but recently developed genome sequence modification technology has the potential to accelerate this process. One of these new breeding technologies relies on an RNA-directed DNA nuclease (CRISPR/Cas9) to cut the genomic DNA, *in vivo*, to facilitate the deletion or insertion of sequences. This sequence specific targeting is determined by guide RNAs (gRNAs). However, choosing an optimum gRNA sequence has its challenges. Almost all current gRNA design tools for use in plants are based on data from experiments in animals, although many allow the use of plant genomes to identify potential off-target sites. Here, we examine the predictive uniformity and performance of eight different online gRNA-site tools. Unfortunately, there was little consensus among the rankings by the different algorithms, nor a statistically significant correlation between rankings and *in vivo* effectiveness. This suggests that important factors affecting gRNA performance and/or target site accessibility, in plants, are yet to be elucidated and incorporated into gRNA-site prediction tools.

## Introduction

Classical plant breeding has dramatically increased the quality and yield of crops for human consumption. However, the cost, time and labour-intensity of crossing and backcrossing and the fact that some crops are vegetatively propagated and essentially sterile (e.g. banana) has driven the development of alternative technologies for crop improvement. One of these is gene editing. When chromosomal DNA is cleaved or broken within a living cell, it is repaired by the endogenous mechanisms of non-homologous end joining (NHEJ) or homology directed repair (HDR) and these processes can be exploited to debilitate, modify or insert genes in the genome [1]. The location of the editing is determined by the location of the double stranded (ds) DNA break which, in turn, can be directed by a sequence-specific nuclease. Zinc finger nucleases (ZFNs) and transcription activator-like effector nucleases (TALENs) were first employed for this purpose but they have been superseded by the CRISPR/Cas nuclease which is directed to its target by a gRNA [1–3]. For the most commonly used CRISPR nuclease, *Streptococcus pyogenes* Cas9 (SpCas9), the gRNA comprises a 20 nt spacer sequence complementary to the DNA target, a 3 nt protospacer-adjacent motif (PAM) of NGG, and a ~70 nt sequence that binds to the nuclease protein. The 20 nt sequence determines the target of the nuclease, but the site must be adjacent to the triplet motif NGG in the genomic context. As there are usually many occurrences of a GG motif in a gene sequence, there are often many sites from which to choose when designing an edit-locating gRNA. The challenge is to predict which gRNA is likely to be the best.

Several different algorithms have been developed for use as online tools to rank the predicted effectiveness of gRNAs. A number of these algorithms were developed based on the assessment of many thousands of gRNAs targeting genes in human or mouse genomes. The online tools generally give a potency score for the candidate gRNA and an option for it to be rejected if it has the potential to direct cleavage in the genome outside of the target. In mammalian systems, the prediction of off-targeting is very important. However, in plants, this is possibly less crucial than the prediction of gRNA sequences that direct efficient Cas9 cleavage of target genes because, in non-vegetatively propagated crops, off-target edits can be easily removed by backcrossing and selection. There are few plant-specific gRNA design tools but the algorithms they employ are based on results from tests in animals. Therefore, we assessed the potency of a range of gRNAs, directing Cas9 against a number of genes in a range of plant species. We then compared the results of these experiments with the predictions from eight different online tools and found that there was no significant correlation between the predicted and observed efficiencies of the gRNA to direct editing.

## Materials and methods

### Construction of CRISPR/Cas9 constructs

The 2X35S-Cas9-NOSt cassette, containing human codon optimised Cas9 (hCas9), was excised from pICH47742::2x35S-5’UTR-hCas9(STOP)-NOSt [4] using restriction enzymes, ApaI and PmeI (New England Biolabs, NEB), and ligated into pORE03 [5] to generate pCas9. The sequence of *A*.*thaliana* small nuclear RNA (snRNA) Pol III promoter with sites for BsaI cloning was ordered as a gene block from IDT (https://www.idtdna.com) and ligated into the unique MluI site of pCas9 to generate pCas9-U3. The second construct, pCas9B contained the 2X CaMV 35S promoter directing expression of a hCas9 with N- and C-terminal nuclear localisation signals (NLS) and a NOSt. hCas9 was used because, in our experience, human codon optimised reporter genes (such as eGFP) work very effectively in a range of plant species and avoids testing monocot-optimised Cas9 in dicots and *vice-versa*. The cassette was assembled in pCAMBIA1300 (CAMBIA, Canberra, Australia) using a Gibson cloning strategy (NEB) according to the manufacturer’s specifications.

The versatile tRNA-gRNA cassette as described by [6] was used for the ease of cloning one or more gRNAs into pCas9 plasmids. A list of DNA oligos used for assembly of gRNAs and for amplification of genic regions for Cas9 editing are provided in S1 Data. Assembly of the TBSV p19 (pJL3:P19) construct was previously described in Lindbo [7] and the TYLCV V2 construct (pCW197) was described in Naim et al., [8]. p2X35S-NanoLuc-NOSt construct was prepared by digesting pCas9 with PspXI and PmeI (NEB) and the backbone ligated to NanoLuc-NOSt cassette, ordered as a gene block (IDT), using Gibson Assembly. Sequences of the Pol III promoters, NanoLuc and the two hSpCas9 are provided in S1 Data. Vectors were mobilised into *Agrobacterium tumefaciens* strain GV3101 or AGL1 for transient and stable transformations.

### Stable transformations and plant regeneration

*A*.*thaliana* was transformed with floral dip techniques [9] with growth conditions and selection performed as previously described [10]. For transformation of *N*.*benthamiana* and *N*.*tabacum*, leaves of 4–5 week old plants grown on soil (16 hr light period at 24°C) were infiltrated with culture(s) of agrobacterium (GV3101) carrying Cas9 plasmid with and without a viral silencing-suppressor (p19 or V2). Leaf infiltrations were performed as previously described [8] and leaves containing a silencing-suppressor were harvested 4–5 days post infiltration (dpi) for analysis of editing. Each analysis was performed with 3–10 biological replicates. Leaves only infiltrated with Cas9 plasmids were used for stable transformation and sterilised in the following steps: infiltrated leaves were dipped in 100% ethanol for 30 sec, washed in sterile water, transferred to 0.6% hypochlorite solution for 20 min and rinsed in sterile water 3 times. Sterile leaves were then excised in 1 cm^2^ pieces and placed on solid MS media (MS104 containing 4.43 g/L MS powder (Sigma-Aldrich M5524), 30 g/L sucrose, 1 mg/L 6-Benzylaminopurine (Sigma-Aldrich B3408), 0.1 mg/L 1-Naphthaleneacetic acid (Sigma-Aldrich N1641), 200 mg/L timentin (Gold Biotechnology N1024944) and 0.5 mL/L PPM (Austratec P820)) for two weeks. Explants were then moved to MS104 containing appropriate selection (200 mg/L kanamycin or 25 mg/L hygromycin) and placed on fresh media every 14 days until shoots were approximately 1–2 cm tall. Shoots were then excised and placed on root initiation media (halved MS powder and sucrose, 0.5 mg/L Indole-3-butyric acid (Sigma-Aldrich I5386), 150 mg/L timentin, 250 mg/L cefotaxime (Gold Biotechnology N1033414), 0.5 mL/L PPM and 100 mg/L kanamycin or 12.5 mg/L hygromycin).

*M*.*acuminata* Cavendish cv. Williams (AAA subgroup) embryogenic cell suspensions were used for transformation and plants regenerated for analysis as previously described by Naim et al., [11]. *O*.*sativa* cv. Niponbarre transgenic plants were generated by the methodology previously described in Sallaud et al., [12]. Agrobacterium-mediated transformation of *O*.*sativa* embryonic calli was conducted with the AGL1 strain. Selection of transformation events was conducted on 50 mg/L of hygromycin included in R2S to RN media [12]. Each individual transgenic event was genotyped by sequencing at the transition of RN media to P media.

### Sequence analysis of CRISPR/Cas9 modified genes

Total DNA from *A*.*thaliana*, *N*.*benthamiana* and *N*.*tabacum* was extracted from approximately 0.5 cm^2^ leaf tissue and ground in 100 uL solution of 50 mM Tris and 0.1 mM EDTA followed by heating at 95°C for five min, ice for 1 min and centrifugation at 14000 rpm for 2 min. PCR was set up using 1.5 uL of the extract as template in 2X 2G Robust HotStart ReadyMix (KAPA Biosystems) with gene specific primers. The PCR cycle conditions were as follows: an initial denaturation step at 95°C for 3 min, 35 cycles of 95°C for 15 sec, 60°C for 15 sec, and 72°C for 15 sec, followed by a final extension at 72°C for 3 min. The resulting PCR products were electrophoresed on a 1% TAE agarose gel and bands excised and cloned into pGEM^®^-T Easy (Promega) according to the manufacturer’s specifications or sequenced directly. Sanger sequencing of 6–12 pGEM clones per independent event, direct sequencing of PCR product and analysis of editing in *M*.*acuminata* events were performed as described previously in Naim *et al*., [11]. Products were Sanger sequenced at QUT CARF and analysed using Geneious R11 (http://www.geneious.com, [13]).

### Online tools and settings

Eight online tools were used to predict and rank the efficiencies of gRNAs targeting sites These were: CRISPRko [14], WU-CRISPR [15], CRISPOR [16], Benchling [14], CCTop [17], sgRNA scorer 2.0 [18], CRISPR-P [19] and Cas-Designer [20]. The prediction tool CRISPOR uses two different algorithms [14, 21] and the rankings of both were assessed and labelled as CRISPOR-M and CRISPOR-D, respectively. The genome assemblies used for evaluating off-target sites in CRISPOR, Benchling, CCTop, CRISPR-P and Cas-Designer were: TAIR10 for *AtmiR166a*, Rice Phytozome V9, *Rice Japonica* and Rice OSV4 for *OsVIT1* and *OsIRO3*, *Musa acuminata Malaccensis*, *Musa acuminata* asm31385v2, *Rice Japonica* and *Musa acuminata* MA1 for *MaPDS* and *MaRDR1*, and Niben101 for *NanoLuc*, *NbRDR1*, *NbRDR2*, *NbRDR6* and *NbFAD2*. The gRNA rankings determined by CRISPR-P for *NanoLuc*, *NbRDR2*, *NbRDR6* and *NtRDR1* were generated without the use of a genome sequence because the offered *N*.*benthamiana* assembly lacks the target gene sequences and no assembly available is offered for *N*.*tabacum*; no rankings are produced for genes missing in draft assembly.

## Results

### Editing a transgene in *Nicotiana benthamiana* by agroinfiltration of a standard Cas9 construct

**Fig 1:**
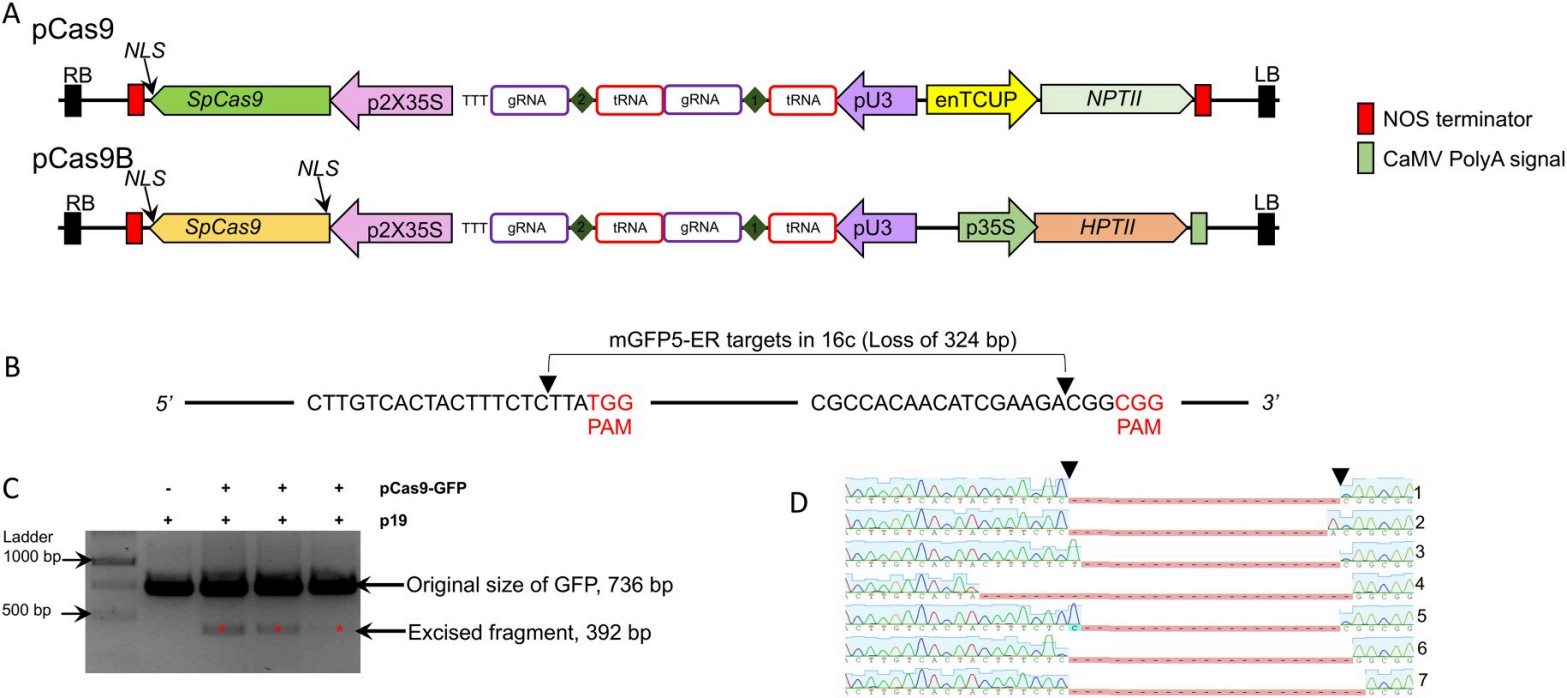
**(A)** Schematic of pCas9 plasmids used in this study. Although two gRNAs are shown for each construct only a single gRNA was used in the transient NanoLuc experiment. **(B)** Schematic mGFP5-ER sequence in transgenic 16c transgenic line used for CRISPR/Cas9 driven gene editing. The two target sites used for gRNA binding are shown and arrows show the predicted site of double strand break by SpCas9. **(C)** Gel image of PCR fragments of a representative leaf infiltrated with p19 and three other leaves infiltrated with pCas9-GFP and p19. The bands denoted with red asterisk on the image show evidence of dropout between the two gRNA target sites. **(D)** Sanger sequence trace of pGEM clones carrying the smaller PCR fragment (indicated with red asterisk in **C**). The arrow indicates the region of Cas9 induced dsDNA break.

A standard construct, pCas9-GFP (Fig 1A), encoding a SpCas9 protein under the control of a double 35S promoter and two gRNAs from the tRNA delivery system [6], was tested for its ability to edit the integrated mGFP5-ER reporter gene in transgenic line 16c of *N*.*benthamiana* [22, 23]. The tRNA system was used to express more than one gRNA from a single Pol III promoter. For ease of detection, gRNAs were designed to guide cleavage at two locations, thereby producing a 344 bp deletion in the transgene (Fig 1B). The sites were chosen on the basis of their distances apart rather than by an online prediction tool. Leaves of 4.5 week-old 16c plants were infiltrated with a cocktail of two agrobacterium cultures: one harbouring the pCas9-GFP construct and the other containing a viral silencing-suppressor construct, expressing the *Tomato bushy stunt virus* (TBSV) *p19* gene [24]. DNA was extracted from the infiltrated patch 5 days post infiltration (dpi) and assayed for the expected deletion by PCR with primers flanking the targeted region. Migration of the products in an agarose gel (Fig 1C) gave bands of approximately the expected sizes for the unedited genomic region (736 nt) and for the region after the edited deletion (392 nt). Cloning and sequencing a population of molecules from the faster migrating band confirmed the expected deletion, although more than 50% showed a further loss of some nucleotides adjacent to the cleavage sites (Fig 1D). More than 50% of molecules from the slower migrating band had small deletions, at either or both of the gRNA target sites, but did not produce a 344bp deletion. These small deletions were presumably made by minor degradation of the cut ends prior to DNA repair by NHEJ.

### Testing the uniformity of cleavage efficiency for a range of target sites in an endogene by transiently expressed Cas9 in *N*.*benthamiana* leaves

**Fig 2:**
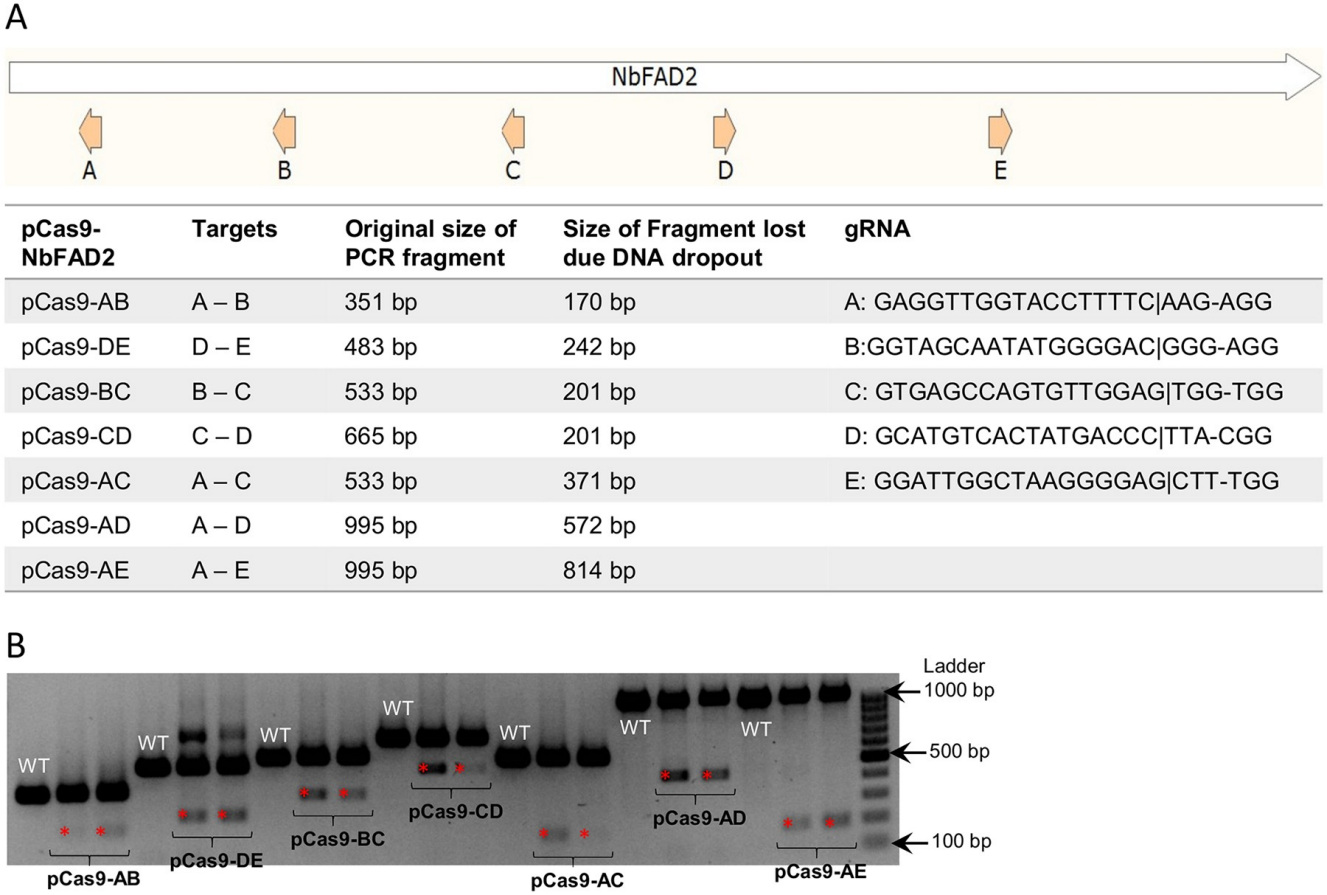
Editing efficiency of *NbFAD2* with combinations of gRNAs targeting various sites of the gene. **(A)** Schematic of *NbFAD2* with annotated five gRNAs used for editing. The table summarises various combinations of gRNAs used for agro-infiltrations, the expected sizes of PCR fragments before editing, the distance between two gRNAs resulting in dropout of DNA and the sequence of the gRNAs. **(B)** Gel image of PCR fragments from a wild-type uninfiltrated sample (WT) and two representative biological replicates of a total of five leaves infiltrated with each pCas9-NbFAD2 plasmid. The replicates shown are representative of the variation seen in leaf infiltration experiments. The bands denoted with a red asterisk on the gel image show evidence of dropout between two gRNAs.

Different sites in the *N*.*benthamiana* endogene, *NbFAD2* (fatty acid desaturase 2, adds a second double bond to oleic acid converting it to linoleic acid), were targeted by gRNAs in various pairwise combinations to evaluate whether they were cleaved with similar efficiency and if the distance between the sites had an effect on excision efficiency (Fig 2A). *NbFAD2* is a single copy gene in *N*.*benthamiana* and we have previously shown successful silencing of this gene using hairpin RNA (hpRNA)-induced RNAi [8]. The sites were chosen because of their relative locations within the gene (~200 nt apart) and deliberately without researcher bias or the aid of a prediction tool. Leaves of 4.5 week old *N*.*benthamiana* plants were agroinfiltrated with constructs similar to pCas9-GFP but with the gRNA pairs targeting *NbFAD2*. Based on the intensities of PCR amplicons representing CRISPR/Cas9-mediated dropouts, all gRNA combinations were effective (Fig 2B). However, different sites appeared to be cleaved at different efficiencies; gRNA pairs DE, BC and AD were the most efficient combinations. In addition, there was no evidence that the size of predicted dropout correlated with editing efficiency (Fig 2B).

### Testing online tools for prediction of gRNA efficiency using a transient assay

**Fig 3:**
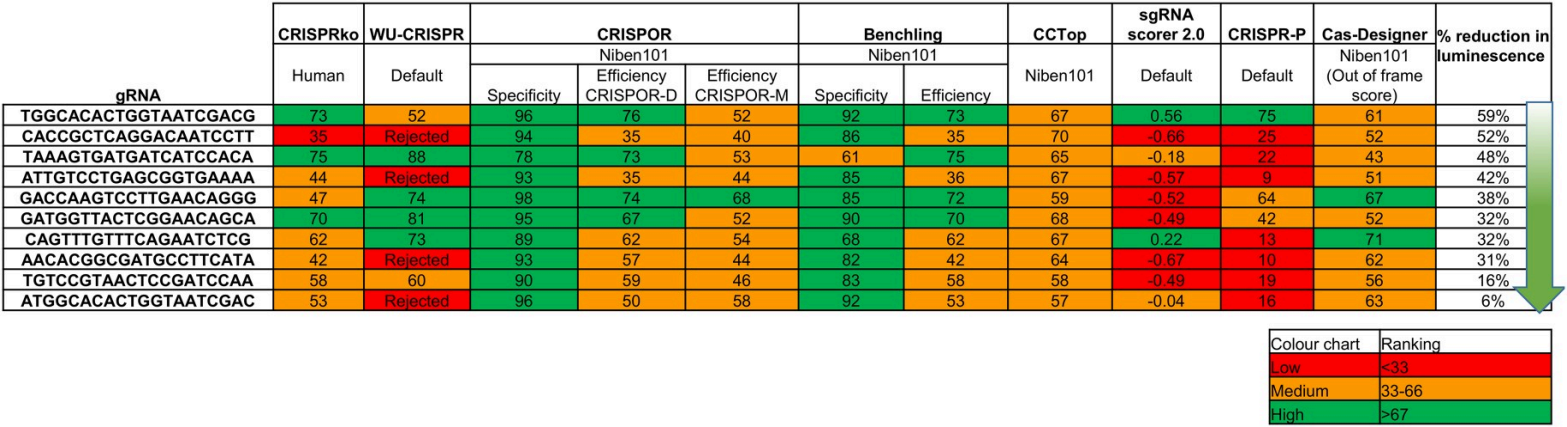
Summary of predicted gRNA rankings vs. observed percent reduction in luminescence of NanoLuc, measured 4 dpi in three biological replicates. NanoLuc luminescence measured in leaves infiltrated with p2X35S-NanoLuc-NOSt, p19 and a NanoLuc editing construct. Percentage reduction of luminescence due to editing of NanoLuc, calculated by setting luminescence of control leaves (infiltrated with p2X35S-NanoLuc-NOSt, p19 and an editing construct targeting an endogene in *N*.*benthamiana*) to 100%. See S1 Data for luminescence measurements. Each gRNA ranking obtained from the online gRNA prediction tools is colour coded: red, orange and green depict low, medium and high editing efficiency, respectively.

Eight online tools were used to predict and rank the efficiencies of ten different gRNAs targeting sites in the high turnover Nano-luciferase (NanoLuc^®^ Promega) reporter gene. The short half-life of NanoLuc makes the assay a measure of gRNA-directed editing efficiency. The NanoLuc sequence was submitted to the following online tools: CRISPRko [14], WU-CRISPR [15], CRISPOR [16], Benchling [14], CCTop [17], sgRNA scorer 2.0 [18], CRISPR-P [19] and Cas-Designer [20]. The prediction tool CRISPOR uses two different algorithms [14, 21] while CRISPOR, Benchling, CCTop and Cas-Designer use the draft *N*.*benthamiana* genome sequence to reject gRNAs with off target binding. On the contrary, CRISPRko is pre-set to use the human genome sequence for off-target rejection, but WU-CRISPR, sgRNA scorer 2.0 and CRISPR-P were given species-independent settings because rankings are not provided for genes not present in the draft genome assembly. The gRNA ranking provided by these algorithms are summarised in Fig 3. The ten gRNA sequences were cloned into pCas9-U3 to generate ten independent editing constructs and these were co-agroinfiltrated with the NanoLuc reporter construct (p2X35S-NanoLuc-NOSt) and p19 into *N*.*benthamiana* leaves. The editing constructs contained a single gRNA. The NanoLuc luminescence readings obtained from mock controls (p2X35S-NanoLuc-NOSt, p19 and an editing construct targeting an endogene in *N*.*benthamiana*) were set to 100% and, in treatments containing pCas9 targeting NanoLuc, the degree of reduction was calculated as percentage reduction from this standard (Fig 3). Raw NanoLuc luminescence measurements are provided in S1 Data. Simple linear regression analysis of these NanoLuc luminescence measurements with the gRNA ranking values showed no statistical correlation for any of the online tool predictions.

### Testing online tools for prediction of gRNA efficiency in stable transgenic lines

**Fig 4:**
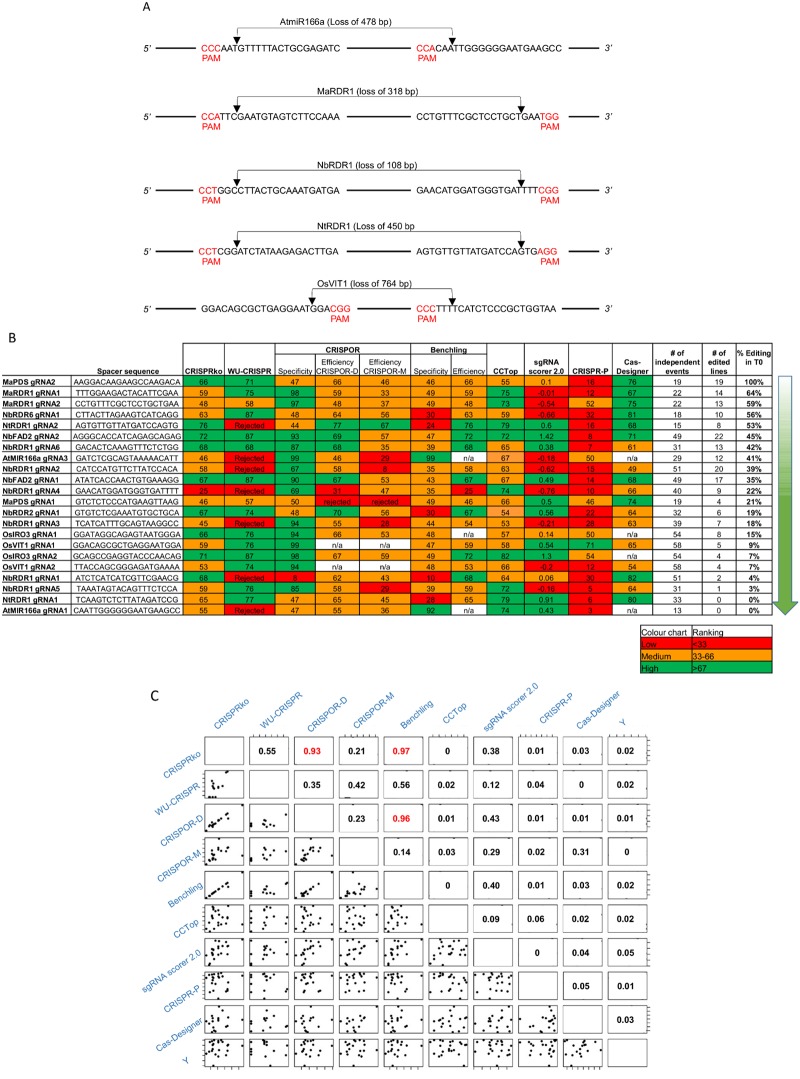
**(A)** Schematic of a representative gene from each plant species used for CRISPR/Cas9 driven gene editing. The two target sites used for gRNA binding are shown and arrows indicate the predicted sites of the double strand breaks made by SpCas9. **(B)** Summary of predicted gRNA rankings versus percentage of observed editing for 22 gRNAs targeting genes in various plant species. The observed rankings are a percentage of edited T0 lines found in an average of 35 independent events analysed for each gRNA. Each gRNA ranking obtained from the online gRNA prediction tools is colour coded: red, orange and green depict low, medium and high editing efficiency, respectively. **(C)** Pairwise scatterplots and R^2^ values between the predicted and observed rankings. “Y” is the observed rankings.

To complement the transient assay experiments, 8 endogenes (*MaPDS*, *Ma/Nb/NtRDR1*, *NbRDR2*, *NbRDR6*, *NbFAD2*, *AtmiR166a*, *OsVIT1* and *OsIRO3*) across 5 different species (*Arabidopsis thaliana*, *Musa acuminata*, *N*.*benthamiana*, *Nicotiana tabacum* and *Oryza sativa*) were modified using CRISPR/Cas9 (Fig 4A). In each case, transgenic plants were generated containing a stably integrated cassette encoding Cas9 and two gRNAs targeting the gene. On average 35 independent transgenic lines were produced per species. The rankings of the gRNAs were determined by the same online tools used for the NanoLuc experiment (Fig 4B).

Comparing the percentage of edited transformants (per site, per species) with the predicted ranking for the gRNAs, using linear and non-parametric regression analyses, showed no significant positive correlation between the predicted rankings and the editing frequencies measured in our experiments (Fig 4B & S1 Data). The plots showed each predicted ranking against what was observed with a line of best fit from the estimated simple linear regression model (S1 Data). For each predictor, there was no evidence against the null hypothesis that the slope is equal. For comparison, a bootstrap analysis was also performed and all 95% bootstrap intervals for each estimated slope parameter included zero, suggesting that there is no evidence of a linear relationship between the predicted and observed rankings. Given the small sample size of this study, a power analysis was also conducted to determine the size of correlations these results were applicable to. This revealed that the sample size had statistical power (>80% at the 5% significance level) to reject high correlations (>0.75) for all predictors except WU-CRISPR and to conclude that there is no evidence that the predicted rankings are highly correlated with the observed rankings. There was no significant positive correlation between the WU-CRISPR rankings and observed editing frequencies, but with reduced statistical power due to 7 missing data points.

Despite the inability of the prediction programs to identify highly susceptible or highly resistant cleavage sites, we often observed in both transient and stable editing experiments that one of the two targeted sites within the gene was much more susceptible than the other. Three extreme examples from stable editing experiments were in *MaPDS*, *NtRDR1*, and *AtmiR166a*. The target 2 site in *MaPDS* was extremely susceptible (100%) and target 1 sites of both *NtRDR1* and *AtmiR166a* were extremely resistant (0%) whereas their partner sites had average susceptibility (21, 53 and 41%, respectively) (Fig 4B). This suggests that all sites are not equally amenable to cleavage but current algorithms are, as yet, unable to accurately discern the susceptible from the resistant. Further evidence of this prediction inaccuracy is that pairwise comparisons of the rankings of the 22 gRNAs by the different programs showed that only CRISPko, CRISPOR and Benchling had positive relationships with R^2^ values of over 60% (Fig 4C).

## Discussion

CRISPR/Cas9 gene editing is a powerful technology for biological research and rapid trait generation in plants. Its precision confers many advantages over the random mutagenesis obtained from chemical mutagens, transposons, or T-DNAs. It also has the potential to be much faster than traditional plant breeding for generating both “knock-out” and “knock-in” traits. A major consideration, when using this system, is the selection of the best gRNA(s) for the purpose. Two important factors when making this selection are the potency of the gRNA (ie how effective it is at guiding efficient cleavage) and the potential to cause “off-target” effects [15]. There are many gRNA selection tools designed for use in animals but very few are intended solely for plants. For example, of the18 programs identified in GM Crops and Food [25], only 2 are plant specific, the rest are intended for cross kingdom application or for use in animals alone. The CGAT program [25] is plant-specific but provides assessment for only 6 plant genomes. The other plant-based design tool, CRISPR-P (which we included in our study), offers genomes for 49 plant species [19], but even this program incorporates design rules based on results from mammalian cell experiments [14]. Furthermore, some of the plant genome sequences in CRISPR-P may not be useful because the target gene sequence is absent from the assembly (e.g. *NbRDR2* and *NbRDR6* which are not available in the offered version for *N*.*benthamiana)*. Nevertheless, the rules designed for gRNAs in animals, may reflect desirable intrinsic features that are equally applicable in plants. In brief, the eight tools we examined to rank our gRNAs use rules, to a greater or lesser extent, that favour a G at −1 and −2 and a C or T at -1, -14 and -17 and avoid an A at -1 and a T at +4/−4 proximal to the PAM. They also avoid a C at the cleavage site, favour an overall GC content between 40–60%, and avoid ending the gRNA with a U or C, due to the formation of disruptive internal secondary structures of the gRNA [15, 26–28]. These gRNA design programs have been helpful in animal genome editing, but they all failed to give potency predictions that significantly correlated with our measured editing efficiencies in plants. Furthermore, there was very little consensus among the programs in their predicted gRNA rankings. A component of this might be that some programs reject gRNAs due to predicted off-target effect, while others do not take this into account or use an inappropriate reference genome sequence (eg. CRISPRko which uses the human genome). However, CRISPOR-D, CRISPOR-M, Benchling, CCTop and Cas-Designer, were all directed to use species specific genomic sequences for this purpose, but only the rankings by CRISPOR-D and Benchling correlated with an R^2^ value >60%. Indeed, CCTop rankings negatively correlated (R = -24%) with those of Cas-Designer, and CRISPRko. Only three programs (CRISPRko, CRISPOR-D and Benchling) had a high degree of inter-ranking consensus (R^2^ = 93–97%) and this is probably because they are all based on data from the same study (Doench et al., [14]. Taken together, our results and results reported by others suggest that almost all gRNA sites in plant genomes are susceptible to at least some degree of Cas9 cleavage, but none of the online prediction programs, that we examined, were very helpful in either avoiding less cleavable sites or selecting highly susceptible ones. It seems that choosing to introduce a knock-out mutation in a gene by targeting Cas9 to the PAM sites in the coding exon to disrupt the function of the resulting protein, is possibly more effective than choosing sites based solely on a gRNA prediction program. Selecting two sites in a gene has the benefit not only of doubling the chances of site cleavage, but also facilitates screening for deletion mutants by PCR.

The conformation of the chromatin surrounding a gRNA/Cas9 target may significantly affect the site’s accessibility. To our knowledge, this is not taken into account in any plant editing algorithms as there is a dearth of knowledge about the epigenetic landscapes of almost all plant genomes. With the advent of ATAC sequencing giving cost-effective genome-wide DNA accessibility profiles [29, 30], this is set to improve. A further consideration is the degree of similarity between the to-be-edited plant genome sequence and the reference sequence. Unless they are near-isogenic, the polymorphisms between the two genomes may well complicate off-target predictions. However, the rapid adoption of CRISPR/Cas to edit plant genomes, the dramatic increase in the quality and spectrum of available plant genome assemblies, the increased breadth of pan-genomic sequencing and the collection of more information about epigenetic genome landscapes, seem likely to facilitate the production of more sophisticated, resourced and useful gRNA scoring programs in the future.

## Supporting information

S1 Data (DOCX)

S1 Raw images (PDF)
