# Supplementary material for: Are the current gRNA ranking prediction algorithms useful for genome editing in plants?
Source: PLoS One. 2020 Jan 24;15(1):e0227994. doi: 10.1371/journal.pone.0227994 (PMC6980586; doi:10.1371/journal.pone.0227994)
Supplement: S1 Data — (DOCX) [file pone.0227994.s001.docx]

Supplementary Material

**­Are the current gRNA ranking prediction algorithms useful for genome editing in plants?**

Fatima Naim^ab*^, Kylie Shand^a^, Satomi Hayashi^a^, Martin O’Brien^c^, James McGree^d^, Alexander A. T. Johnson^c^, Benjamin Dugdale^a^ and Peter M. Waterhouse^a*^

^a^Centre for Tropical Crops and Biocommodities, Science and Engineering Faculty, Queensland University of Technology, Brisbane, Queensland 4000, Australia

^b^Centre for Crop and Disease Management, School of Molecular and Life Sciences, Curtin University, Bentley, Western Australia 6102, Australia

^c^School of BioSciences, The University of Melbourne, Victoria 3010, Australia

^d^School of Mathematical Sciences, Science and Engineering Faculty, Queensland University of Technology, Brisbane, Queensland 4000, Australia

^*^ Correspondence

Dr Fatima Naim

[fatima.naim@curtin.edu.au](mailto:fatima.naim@curtin.edu.au)

Professor Peter Waterhouse

[peter.waterhouse@qut.edu.au](mailto:peter.waterhouse@qut.edu.au)

**Supplementary Fig 1.** NanoLuc luminescence measured in leaf tissue infiltrated with p2X35S-NanoLuc-NOSt (p35S-NLUC), p19 and with or without an editing construct targeting NanoLuc. Control infiltration contained p2X35S-NanoLuc-NOSt (p35S-NLUC), p19 and an editing construct targeting an endogenous *N.benthamiana* gene.

NanoLuc Luminescence

Infiltration treatments


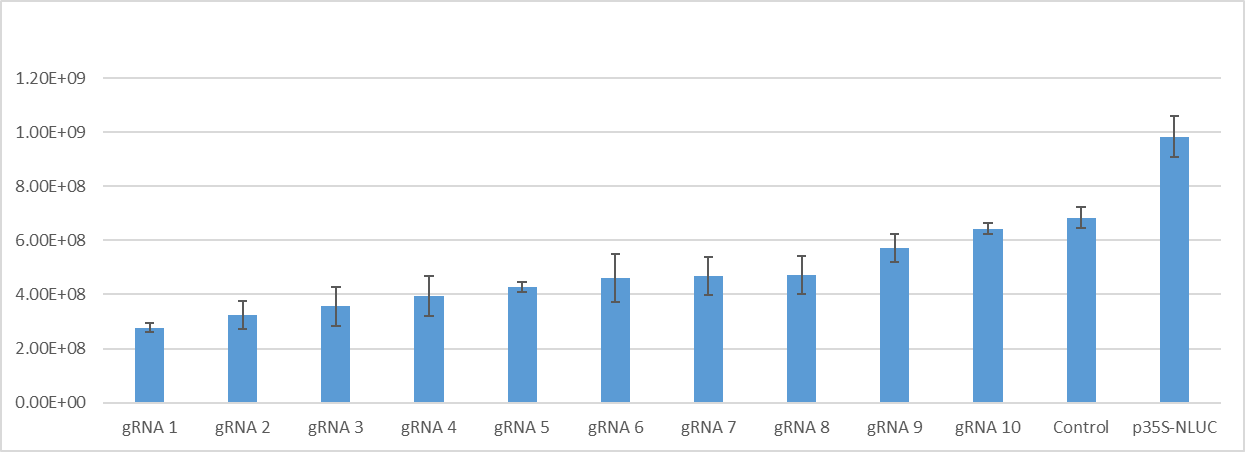


**Supplementary Figure 2.** Statistical analysis of observed Vs predicted rankings for 22 gRNAs targeting genes in various plant species.

- An observed variable (real data, denote as ‘y’);
- 9 predictor variables (from algorithms, denote as x1, x2, ..., x9) (CRISPRko, WU-CRISPR, CRISPOR-D, CRISPOR-M, Benchling, CCTop, sgRNA scorer 2.0, CRISPR-P, Cas-Designer)

1. **Assume linear model appropriate (normally distributed errors)**


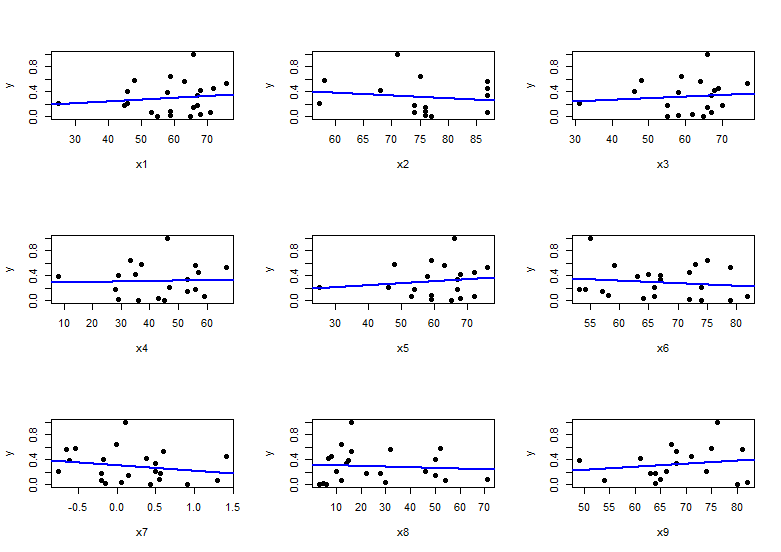


Summary of results for each regression model

|  | Estimate | Std. Error | t-value | Pr(>\|t\|) |
| --- | --- | --- | --- | --- |
| (Intercept) | 0.139079 | 0.294245 | 0.472663 | 0.641571 |
| X1 | 0.002712 | 0.004881 | 0.555693 | 0.584584 |
|  |  |  |  |  |
| (Intercept) | 0.62828 | 0.628726 | 0.99929 | 0.335892 |
| X2 | -0.00408 | 0.008285 | -0.49213 | 0.630835 |
|  |  |  |  |  |
| (Intercept) | 0.167104 | 0.377076 | 0.443157 | 0.66324 |
| X3 | 0.002651 | 0.006138 | 0.431868 | 0.671265 |
|  |  |  |  |  |
| (Intercept) | 0.293294 | 0.204393 | 1.434946 | 0.169445 |
| X4 | 0.0008 | 0.004522 | 0.176864 | 0.861705 |
|  |  |  |  |  |
| (Intercept) | 0.136155 | 0.333126 | 0.40872 | 0.687848 |
| X5 | 0.003006 | 0.005431 | 0.553568 | 0.587082 |
|  |  |  |  |  |
| (Intercept) | 0.579249 | 0.450865 | 1.28475 | 0.213561 |
| X6 | -0.00417 | 0.006677 | -0.62511 | 0.538969 |
|  |  |  |  |  |
| (Intercept) | 0.316986 | 0.057955 | 5.469549 | 2.36E-05 |
| X7 | -0.09357 | 0.095022 | -0.9847 | 0.336536 |
|  |  |  |  |  |
| (Intercept) | 0.324054 | 0.092918 | 3.487506 | 0.002321 |
| X8 | -0.00096 | 0.002907 | -0.33116 | 0.743971 |
|  |  |  |  |  |
| (Intercept) | -0.01572 | 0.508833 | -0.03089 | 0.975739 |
| X9 | 0.00508 | 0.007399 | 0.686509 | 0.502221 |

There is no evidence against there being no linear relationship between these variables. That is, there was no evidence against beta1 (slope) equaling 0.

**Supplementary Table 1.** List of DNA oligos used for assembly of gRNAs corresponding to various targeted genes in this study.

| **Stable** | **gRNA sequence** | **Oligo Sense** | **Oligo Antisense** |
| --- | --- | --- | --- |
| Generic AtU3-BsaI primer | n/a | ATGTACTCGATCAGGTCTCAGGTCAACAAAGC | n/a |
| Generic OsU3-BsaI primer | n/a | ATGTACTCGATCAGGTCTCATGGCAACAAAGC | n/a |
| Generic gRNA-BsaI primer | n/a | n/a | TAGGCATGACTATGGTCTCTGTAGTAAAAAAAAAAGCAC |
| NanoLuc gRNA 1 | TGGCACACTGGTAATCGACG | TAGGTCTCCTGGTAATCGACGGTTTTAGAGCTAGAAATAG | TAGGTCTCAACCAGTGTGCCATGCACCAGCCGGGAATC |
| NanoLuc gRNA 2 | CACCGCTCAGGACAATCCTT | TAGGTCTCCAGGACAATCCTTGTTTTAGAGCTAGAAATAG | TAGGTCTCATCCTGAGCGGTGTGCACCAGCCGGGAATC |
| NanoLuc gRNA 3 | TAAAGTGATGATCATCCACA | TAGGTCTCCTGATCATCCACAGTTTTAGAGCTAGAAATAG | TAGGTCTCAATCATCACTTTATGCACCAGCCGGGAATC |
| NanoLuc gRNA 4 | ATTGTCCTGAGCGGTGAAAA | TAGGTCTCCGAGCGGTGAAAAGTTTTAGAGCTAGAAATAG | TAGGTCTCAGCTCAGGACAATTGCACCAGCCGGGAATC |
| NanoLuc gRNA 5 | GACCAAGTCCTTGAACAGGG | TAGGTCTCCCCTTGAACAGGGGTTTTAGAGCTAGAAATAG | TAGGTCTCAAAGGACTTGGTCTGCACCAGCCGGGAATC |
| NanoLuc gRNA 6 | GATGGTTACTCGGAACAGCA | TAGGTCTCCCTCGGAACAGCAGTTTTAGAGCTAGAAATAG | TAGGTCTCACGAGTAACCATCTGCACCAGCCGGGAATC |
| NanoLuc gRNA 7 | CAGTTTGTTTCAGAATCTCG | TAGGTCTCCTTCAGAATCTCGGTTTTAGAGCTAGAAATAG | TAGGTCTCATGAAACAAACTGTGCACCAGCCGGGAATC |
| NanoLuc gRNA 8 | AACACGGCGATGCCTTCATA | TAGGTCTCCGATGCCTTCATAGTTTTAGAGCTAGAAATAG | TAGGTCTCACATCGCCGTGTTTGCACCAGCCGGGAATC |
| NanoLuc gRNA 9 | TGTCCGTAACTCCGATCCAA | TAGGTCTCCACTCCGATCCAAGTTTTAGAGCTAGAAATAG | TAGGTCTCAGAGTTACGGACATGCACCAGCCGGGAATC |
| NanoLuc gRNA 10 | ATGGCACACTGGTAATCGAC | TAGGTCTCCCTGGTAATCGACGTTTTAGAGCTAGAAATAG | TAGGTCTCACCAGTGTGCCATTGCACCAGCCGGGAATC |
| AtMir166a target 1 | CAATTGGGGGGAATGAAGCC | TAGGTCTCCGGGAATGAAGCCGTTTTAGAGCTAGAAATAG | TAGGTCTCATCCCCCCAATTGTGCACCAGCCGGGAATC |
| AtMir166a target 3 | GATCTCGCAGTAAAAACATT | TAGGTCTCCAGTAAAAACATTGTTTTAGAGCTAGAAATAG | ATGGTCTCATACTGCGAGATCTGCACCAGCCGGGAATC |
| MaPDS target 1 | GTCTCTCCCATGAAGTTAAG | TAGGTCTCCCATGAAGTTAAGGTTTTAGAGCTAGAAATAG | TAGGTCTCACATGGGAGAGACTGCACCAGCCGGGAATC |
| MaPDS target 2 | AAGGACAAGAAGCCAAGACA | TAGGTCTCCGAAGCCAAGACAGTTTTAGAGCTAGAAATAG | ATGGTCTCACTTCTTGTCCTTTGCACCAGCCGGGAATC |
| MaRDR1 target 1 | TTTGGAAGACTACATTCGAA | TAGGTCTCCACTACATTCGAAGTTTTAGAGCTAGAAATAG | TAGGTCTCATAGTCTTCCAAATGCACCAGCCGGGAATC |
| MaRDR1 target 2 | CCTGTTTCGCTCCTGCTGAA | TAGGTCTCCGCTCCTGCTGAAGTTTTAGAGCTAGAAATAG | ATGGTCTCAGAGCGAAACAGGTGCACCAGCCGGGAATC |
| mGFP5-ER target 1 | CTTGTCACTACTTTCTCTTA | TAGGTCTCCTACTTTCTCTTAGTTTTAGAGCTAGAAATAG | TAGGTCTCAAGTAGTGACAAGTGCACCAGCCGGGAATC |
| mGFP5-ER target 2 | CGCCACAACATCGAAGACGG | TAGGTCTCCCATCGAAGACGGGTTTTAGAGCTAGAAATAG | ATGGTCTCAGATGTTGTGGCGTGCACCAGCCGGGAATC |
| NbFAD2 target 1 | ATATCACCAACTGTGAAAGG | TAGGTCTCCAACTGTGAAAGGGTTTTAGAGCTAGAAATAG | TAGGTCTCAAGTTGGTGATATTGCACCAGCCGGGAATC |
| NbFAD2 target 2 | AGGGCACCATCAGAGCAGAG | TAGGTCTCCATCAGAGCAGAGGTTTTAGAGCTAGAAATAG | ATGGTCTCATGATGGTGCCCTTGCACCAGCCGGGAATC |
| NbFAD2 target A | GAGGTTGGTACCTTTTCAAG | TAGGTCTCCTACCTTTTCAAGGTTTTAGAGCTAGAAATAG | TAGGTCTCAGGTACCAACCTCTGCACCAGCCGGGAATC |
| NbFAD2 target B | GGTAGCAATATGGGGACGGG | TAGGTCTCCTATGGGGACGGGGTTTTAGAGCTAGAAATAG | ATGGTCTCACATATTGCTACCTGCACCAGCCGGGAATC |
| NbFAD2 target C | GTGAGCCAGTGTTGGAGTGG | TAGGTCTCCGTGTTGGAGTGGGTTTTAGAGCTAGAAATAG | TAGGTCTCAACACTGGCTCACTGCACCAGCCGGGAATC |
| NbFAD2 target D | GCATGTCACTATGACCCTTA | TAGGTCTCCCTATGACCCTTAGTTTTAGAGCTAGAAATAG | TAGGTCTCAATAGTGACATGCTGCACCAGCCGGGAATC |
| NbFAD2 target E | GGATTGGCTAAGGGGAGCTT | TAGGTCTCCTAAGGGGAGCTTGTTTTAGAGCTAGAAATAG | ATGGTCTCACTTAGCCAATCCTGCACCAGCCGGGAATC |
| NbRDR1 target 1 | ATCTCATCATCGTTCGAACG | TAGGTCTCCATCGTTCGAACGGTTTTAGAGCTAGAAATAG | TAGGTCTCACGATGATGAGATTGCACCAGCCGGGAATC |
| NbRDR1 target 2 | CATCCATGTTCTTATCCACA | TAGGTCTCCTTCTTATCCACAGTTTTAGAGCTAGAAATAG | ATGGTCTCAAGAACATGGATGTGCACCAGCCGGGAATC |
| NbRDR1 target 3 | TCATCATTTGCAGTAAGGCC | TAGGTCTCCTGCAGTAAGGCCGTTTTAGAGCTAGAAATAG | TAGGTCTCATGCAAATGATGATGCACCAGCCGGGAATC |
| NbRDR1 target 4 | GAACATGGATGGGTGATTTT | TAGGTCTCCATGGGTGATTTTGTTTTAGAGCTAGAAATAG | ATGGTCTCACCATCCATGTTCTGCACCAGCCGGGAATC |
| NbRDR1 target 5 | TAAATAGTACAGTTTCTCCA | TAGGTCTCCAAGTTTCTCTGGGTTTTAGAGCTAGAAATAG | ATGGTCTCAACTTTGAGTGTCTGCACCAGCCGGGAATC |
| NbRDR1 target 6 | GACACTCAAAGTTTCTCTGG | TAGGTCTCCACAGTTTCTCCAGTTTTAGAGCTAGAAATAG | TAGGTCTCACTGTACTATTTATGCACCAGCCGGGAATC |
| NbRDR2 target 1 | GTGTCTCGAAATGTGCTGCA | TAGGTCTCCAAATGTGCTGCAGTTTTAGAGCTAGAAATAG | ATGGTCTCAATTTCGAGACACTGCACCAGCCGGGAATC |
| NbRDR6 target 1 | CTTACTTAGAAGTCATCAGG | TAGGTCTCCGAAGTCATCAGGGTTTTAGAGCTAGAAATAG | TAGGTCTCACTTCTAAGTAAGTGCACCAGCCGGGAATC |
| NtRDR1 target 1 | TCAAGTCTCTTATAGATCCG | TAGGTCTCCCTTATAGATCCGGTTTTAGAGCTAGAAATAG | TAGGTCTCATAAGAGACTTGATGCACCAGCCGGGAATC |
| NtRDR1 target 2 | AGTGTTGTTATGATCCAGTG | TAGGTCTCCTATGATCCAGTGGTTTTAGAGCTAGAAATAG | ATGGTCTCACATAACAACACTTGCACCAGCCGGGAATC |
| OsIRO3 target 1 | GGATAGGCAGAGTAATGGGA | TAGGTCTCCAGAGTAATGGGAGTTTTAGAGCTAGAAATAG | TAGGTCTCACTCTGCCTATCCTGCACCAGCCGGGAATC |
| OsIRO3 target 2 | GCAGCCGAGGTACCCAACAG | TAGGTCTCCGGTACCCAACAGGTTTTAGAGCTAGAAATAG | ATGGTCTCATACCTCGGCTGCTGCACCAGCCGGGAATC |
| OsVIT1 target 1 | GGACAGCGCTGAGGAATGGA | TAGGTCTCCCTGAGGAATGGAGTTTTAGAGCTAGAAATAG | TAGGTCTCATCAGCGCTGTCCTGCACCAGCCGGGAATC |
| OsVIT1 target 2 | TTACCAGCGGGAGATGAAAA | TAGGTCTCCGGGAGATGAAAAGTTTTAGAGCTAGAAATAG | ATGGTCTCATCCCGCTGGTAATGCACCAGCCGGGAATC |

**Supplementary Table 2.** List of DNA oligos used for amplification of genic regions for Sanger sequencing.

| **Gene** | **Gene specific Sense** | **Gene Specific Antisense** |
| --- | --- | --- |
| AtMir166a | CATGGCCATAATTTGTTTGC | CTATCGGTCCCCAAAGACCT |
| MaPDS | TTTAATTATCGATTGTAGATAGACC | GAGGGCTG GCACCATGTCTTGGCT |
| MaRDR1 | CGTAATACTGGTTCCCTATATGAAG | GTCACCTGCACCCTGTGAAC |
| mGFP5-ER | GGGCACAAATTTTCTGTCAGTGG | CGCAAGACCGGCAACAGGATTC |
| NbFAD2 | ATGGGAGCTGGTGGTAATATG | CGGCCTGGTGGATTGTTCAAG |
| NbFAD2 | CTTGAACAATCCACCAGGCCG | GCCTCCATTGCATTGTAGTGTG |
| NbFAD2 | GCCTTTAGTGACTACCAGTGG | CCACTGGTAGTCACTAAAGGC |
| NbRDR1 | AAGGGTCGTAGTGACACCAT | CAACATCATGCCTACCGACACTC |
| NbRDR1 | ACATGGATGCTTTCCTGGGG | ACGGCAACTCTACGAGCAAA |
| NbRDR2 | TCTTTTCCTGGCGTTCTCGG | TCGTGCATAAACCTGGCCAT |
| NbRDR6 | CATTCAACGGGGCATGCAAA | TCCAACTCTCCAGTTCTCTCGA |
| NtRDR1 | AAGCCATTCCGAAGAGTTGA | AGTTATTTGGTGCTCCTCGGATC |
| OsIRO3 | ACTAGCAACTGGATTCATCATTGC | TGTGCTATCATCTATCGTGCTACC |
| OsVIT1 | CACTTACCCGCAATTGCACC | GCCTTGGCCATTCCATATGC |

**Sequences:**

AtU3 promoter

CTTATGGCTCAGCCTGTGATGGATAACTGAATCAAACAAATGGCGTCTGGGTTTAAGAAGATCTGTTTTGGCTATGTTGGACGAAACAAGTGAACTTTTAGGATCAACTTCCGTTTATATACGGAGCTTATATCGAGCAATAAGATAAGTGGGCTTTTTATGTAATTTAATGGGCTATCGTCCATATATTCACTAATACCCATGCCCAGTACCCATGTATGCGTTTCATATAAGCTCCTAATTTCTCCCACATCGCTCAAATCTAAACAAATCTTGTTGTATATATAACACTGAGGGAGCACCATTGGTC

OsU3 promoter

AAGGAATCTTTAAACATACGAACAGATCACTTAAAGTTCTTCTGAAGCAACTTAAAGTTATCAGGCATGCATGGATCTTGGAGGAATCAGATGTGCAGTCAGGGACCATAGCACAAGACAGGCGTCTTCTACTGGTGCTACCAGCAAATGCTGGAAGCCGGGAACACTGGGTACGTTGGAAACCACGTGATGTGAAGAAGTAAGATAAACTGTAGGAGAAAAGCATTTCGTAGTGGGCCATGAAGCCTTTCAGGACATGTATTGCAGTATGGGCCGGCCCATTACGCAATTGGACGACAACAAAGACTAGTATTAGTACCACCTCGGCTATCCACATAGATCAAAGCTGATTTAAAAGAGTTGTGCAGATGATCCGTGGC

NanoLuc ORF

ATGGTCTTCACACTCGAAGATTTCGTTGGGGACTGGCGACAGACAGCCGGCTACAACCTGGACCAAGTCCTTGAACAGGGAGGTGTGTCCAGTTTGTTTCAGAATCTCGGGGTGTCCGTAACTCCGATCCAAAGGATTGTCCTGAGCGGTGAAAATGGGCTGAAGATCGACATCCATGTCATCATCCCGTATGAAGGTCTGAGCGGCGACCAAATGGGCCAGATCGAAAAAATTTTTAAGGTGGTGTACCCTGTGGATGATCATCACTTTAAGGTGATCCTGCACTATGGCACACTGGTAATCGACGGGGTTACGCCGAACATGATCGACTATTTCGGACGGCCGTATGAAGGCATCGCCGTGTTCGACGGCAAAAAGATCACTGTAACAGGGACCCTGTGGAACGGCAACAAAATTATCGACGAGCGCCTGATCAACCCCGACGGCTCCCTGCTGTTCCGAGTAACCATCAACGGAGTGACCGGCTGGCGGCTGTGCGAACGCATTCTGGCGTAA

hSpCas9

ATGGACAAGAAGTACTCCATTGGGCTCGATATCGGCACAAACAGCGTCGGCTGGGCCGTCATTACGGACGAGTACAAGGTGCCGAGCAAAAAATTCAAAGTTCTGGGCAATACCGATCGCCACAGCATAAAGAAGAACCTCATTGGCGCCCTCCTGTTCGACTCCGGGGAGACGGCCGAAGCCACGCGGCTCAAAAGAACAGCACGGCGCAGATATACCCGCAGAAAGAATCGGATCTGCTACCTGCAGGAGATCTTTAGTAATGAGATGGCTAAGGTGGATGACTCTTTCTTCCATAGGCTGGAGGAGTCCTTTTTGGTGGAGGAGGATAAAAAGCACGAGCGCCACCCAATCTTTGGCAATATCGTGGACGAGGTGGCGTACCATGAAAAGTACCCAACCATATATCATCTGAGGAAGAAGCTTGTAGACAGTACTGATAAGGCTGACTTGCGGTTGATCTATCTCGCGCTGGCGCATATGATCAAATTTCGGGGACACTTCCTCATCGAGGGGGACCTGAACCCAGACAACAGCGATGTCGACAAACTCTTTATCCAACTGGTTCAGACTTACAATCAGCTTTTCGAAGAGAACCCGATCAACGCATCCGGAGTTGACGCCAAAGCAATCCTGAGCGCTAGGCTGTCCAAATCCCGGCGGCTCGAAAACCTCATCGCACAGCTCCCTGGGGAGAAGAAGAACGGCCTGTTTGGTAATCTTATCGCCCTGTCACTCGGGCTGACCCCCAACTTTAAATCTAACTTCGACCTGGCCGAAGATGCCAAGCTTCAACTGAGCAAAGACACCTACGATGATGATCTCGACAATCTGCTGGCCCAGATCGGCGACCAGTACGCAGACCTTTTTTTGGCGGCAAAGAACCTGTCAGACGCCATTCTGCTGAGTGATATTCTGCGAGTGAACACGGAGATCACCAAAGCTCCGCTGAGCGCTAGTATGATCAAGCGCTATGATGAGCACCACCAAGACTTGACTTTGCTGAAGGCCCTTGTCAGACAGCAACTGCCTGAGAAGTACAAGGAAATTTTCTTCGATCAGTCTAAAAATGGCTACGCCGGATACATTGACGGCGGAGCAAGCCAGGAGGAATTTTACAAATTTATTAAGCCCATCTTGGAAAAAATGGACGGCACCGAGGAGCTGCTGGTAAAGCTTAACAGAGAAGATCTGTTGCGCAAACAGCGCACTTTCGACAATGGAAGCATCCCCCACCAGATTCACCTGGGCGAACTGCACGCTATCCTCAGGCGGCAAGAGGATTTCTACCCCTTTTTGAAAGATAACAGGGAAAAGATTGAGAAAATCCTCACATTTCGGATACCCTACTATGTAGGCCCCCTCGCCCGGGGAAATTCCAGATTCGCGTGGATGACTCGCAAATCAGAAGAGACTATCACTCCCTGGAACTTCGAGGAAGTCGTGGATAAGGGGGCCTCTGCCCAGTCCTTCATCGAAAGGATGACTAACTTTGATAAAAATCTGCCTAACGAAAAGGTGCTTCCTAAACACTCTCTGCTGTACGAGTACTTCACAGTTTATAACGAGCTCACCAAGGTCAAATACGTCACAGAAGGGATGAGAAAGCCAGCATTCCTGTCTGGAGAGCAGAAGAAAGCTATCGTGGACCTCCTCTTCAAGACGAACCGGAAAGTTACCGTGAAACAGCTCAAAGAAGATTATTTCAAAAAGATTGAATGTTTCGACTCTGTTGAAATCAGCGGAGTGGAGGATCGCTTCAACGCATCCCTGGGAACGTATCACGATCTCCTGAAAATCATTAAAGACAAGGACTTCCTGGACAATGAGGAGAACGAGGACATTCTTGAGGACATTGTCCTCACCCTTACGTTGTTTGAAGATAGGGAGATGATTGAAGAACGCTTGAAAACTTACGCTCATCTCTTCGACGACAAAGTCATGAAACAGCTCAAGAGGCGCCGATATACAGGATGGGGGCGGCTGTCAAGAAAACTGATCAATGGGATCCGAGACAAGCAGAGTGGAAAGACAATCCTGGATTTTCTTAAGTCCGATGGATTTGCCAACCGGAACTTCATGCAGTTGATCCATGATGACTCTCTCACCTTTAAGGAGGACATCCAGAAAGCACAAGTTTCTGGCCAGGGGGACAGTCTCCACGAGCACATCGCTAATCTTGCAGGTAGCCCAGCTATCAAAAAGGGAATACTGCAGACCGTTAAGGTCGTGGATGAACTCGTCAAAGTAATGGGAAGGCATAAGCCCGAGAATATCGTTATCGAGATGGCCCGAGAGAACCAAACTACCCAGAAGGGACAGAAGAACAGTAGGGAAAGGATGAAGAGGATTGAAGAGGGTATAAAAGAACTGGGGTCCCAAATCCTTAAGGAACACCCAGTTGAAAACACCCAGCTTCAGAATGAGAAGCTCTACCTGTACTACCTGCAGAACGGCAGGGACATGTACGTGGATCAGGAACTGGACATCAATCGGCTCTCCGACTACGACGTGGATCATATCGTGCCCCAGTCTTTTCTCAAAGATGATTCTATTGATAATAAAGTGTTGACAAGATCCGATAAAAATAGAGGGAAGAGTGATAACGTCCCCTCAGAAGAAGTTGTCAAGAAAATGAAAAATTATTGGCGGCAGCTGCTGAACGCCAAACTGATCACACAACGGAAGTTCGATAATCTGACTAAGGCTGAACGAGGTGGCCTGTCTGAGTTGGATAAAGCCGGCTTCATCAAAAGGCAGCTTGTTGAGACACGCCAGATCACCAAGCACGTGGCCCAAATTCTCGATTCACGCATGAACACCAAGTACGATGAAAATGACAAACTGATTCGAGAGGTGAAAGTTATTACTCTGAAGTCTAAGCTGGTTTCAGATTTCAGAAAGGACTTTCAGTTTTATAAGGTGAGAGAGATCAACAATTACCACCATGCGCATGATGCCTACCTGAATGCAGTGGTAGGCACTGCACTTATCAAAAAATATCCCAAGCTTGAATCTGAATTTGTTTACGGAGACTATAAAGTGTACGATGTTAGGAAAATGATCGCAAAGTCTGAGCAGGAAATAGGCAAGGCCACCGCTAAGTACTTCTTTTACAGCAATATTATGAATTTTTTCAAGACCGAGATTACACTGGCCAATGGAGAGATTCGGAAGCGACCACTTATCGAAACAAACGGAGAAACAGGAGAAATCGTGTGGGACAAGGGTAGGGATTTCGCGACAGTCCGGAAGGTCCTGTCCATGCCGCAGGTGAACATCGTTAAAAAGACCGAAGTACAGACCGGAGGCTTCTCCAAGGAAAGTATCCTCCCGAAAAGGAACAGCGACAAGCTGATCGCACGCAAAAAAGATTGGGACCCCAAGAAATACGGCGGATTCGATTCTCCTACAGTCGCTTACAGTGTACTGGTTGTGGCCAAAGTGGAGAAAGGGAAGTCTAAAAAACTCAAAAGCGTCAAGGAACTGCTGGGCATCACAATCATGGAGCGATCAAGCTTCGAAAAAAACCCCATCGACTTTCTCGAGGCGAAAGGATATAAAGAGGTCAAAAAAGACCTCATCATTAAGCTTCCCAAGTACTCTCTCTTTGAGCTTGAAAACGGCCGGAAACGAATGCTCGCTAGTGCGGGCGAGCTGCAGAAAGGTAACGAGCTGGCACTGCCCTCTAAATACGTTAATTTCTTGTATCTGGCCAGCCACTATGAAAAGCTCAAAGGATCTCCCGAAGATAATGAGCAGAAGCAGCTGTTCGTGGAACAACACAAACACTACCTTGATGAGATCATCGAGCAAATAAGCGAATTCTCCAAAAGAGTGATCCTCGCCGACGCTAACCTCGATAAGGTGCTTTCTGCTTACAATAAGCACAGGGATAAGCCCATCAGGGAGCAGGCAGAAAACATTATCCACTTGTTTACTCTGACCAACTTGGGCGCGCCTGCAGCCTTCAAGTACTTCGACACCACCATAGACAGAAAGCGGTACACCTCTACAAAGGAGGTCCTGGACGCCACACTGATTCATCAGTCAATTACGGGGCTCTATGAAACAAGAATCGACCTCTCTCAGCTCGGTGGAGACAGCAGGGCTGACCCCAAGAAGAAGAGGAAGGTGTGA

hSpCas9B

ATGGACTATAAGGACCACGACGGAGACTACAAGGATCATGATATTGATTACAAAGACGATGACGATAAGATGGCCCCAAAGAAGAAGCGGAAGGTCGGTATCCACGGAGTCCCAGCAGCCGACAAGAAGTACAGCATCGGCCTGGACATCGGCACCAACTCTGTGGGCTGGGCCGTGATCACCGACGAGTACAAGGTGCCCAGCAAGAAATTCAAGGTGCTGGGCAACACCGACCGGCACAGCATCAAGAAGAACCTGATCGGAGCCCTGCTGTTCGACAGCGGCGAAACAGCCGAGGCCACCCGGCTGAAGAGAACCGCCAGAAGAAGATACACCAGACGGAAGAACCGGATCTGCTATCTGCAAGAGATCTTCAGCAACGAGATGGCCAAGGTGGACGACAGCTTCTTCCACAGACTGGAAGAGTCCTTCCTGGTGGAAGAGGATAAGAAGCACGAGCGGCACCCCATCTTCGGCAACATCGTGGACGAGGTGGCCTACCACGAGAAGTACCCCACCATCTACCACCTGAGAAAGAAACTGGTGGACAGCACCGACAAGGCCGACCTGCGGCTGATCTATCTGGCCCTGGCCCACATGATCAAGTTCCGGGGCCACTTCCTGATCGAGGGCGACCTGAACCCCGACAACAGCGACGTGGACAAGCTGTTCATCCAGCTGGTGCAGACCTACAACCAGCTGTTCGAGGAAAACCCCATCAACGCCAGCGGCGTGGACGCCAAGGCCATCCTGTCTGCCAGACTGAGCAAGAGCAGACGGCTGGAAAATCTGATCGCCCAGCTGCCCGGCGAGAAGAAGAATGGCCTGTTCGGAAACCTGATTGCCCTGAGCCTGGGCCTGACCCCCAACTTCAAGAGCAACTTCGACCTGGCCGAGGATGCCAAACTGCAGCTGAGCAAGGACACCTACGACGACGACCTGGACAACCTGCTGGCCCAGATCGGCGACCAGTACGCCGACCTGTTTCTGGCCGCCAAGAACCTGTCCGACGCCATCCTGCTGAGCGACATCCTGAGAGTGAACACCGAGATCACCAAGGCCCCCCTGAGCGCCTCTATGATCAAGAGATACGACGAGCACCACCAGGACCTGACCCTGCTGAAAGCTCTCGTGCGGCAGCAGCTGCCTGAGAAGTACAAAGAGATTTTCTTCGACCAGAGCAAGAACGGCTACGCCGGCTACATTGACGGCGGAGCCAGCCAGGAAGAGTTCTACAAGTTCATCAAGCCCATCCTGGAAAAGATGGACGGCACCGAGGAACTGCTCGTGAAGCTGAACAGAGAGGACCTGCTGCGGAAGCAGCGGACCTTCGACAACGGCAGCATCCCCCACCAGATCCACCTGGGAGAGCTGCACGCCATTCTGCGGCGGCAGGAAGATTTTTACCCATTCCTGAAGGACAACCGGGAAAAGATCGAGAAGATCCTGACCTTCCGCATCCCCTACTACGTGGGCCCTCTGGCCAGGGGAAACAGCAGATTCGCCTGGATGACCAGAAAGAGCGAGGAAACCATCACCCCCTGGAACTTCGAGGAAGTGGTGGACAAGGGCGCTTCCGCCCAGAGCTTCATCGAGCGGATGACCAACTTCGATAAGAACCTGCCCAACGAGAAGGTGCTGCCCAAGCACAGCCTGCTGTACGAGTACTTCACCGTGTATAACGAGCTGACCAAAGTGAAATACGTGACCGAGGGAATGAGAAAGCCCGCCTTCCTGAGCGGCGAGCAGAAAAAGGCCATCGTGGACCTGCTGTTCAAGACCAACCGGAAAGTGACCGTGAAGCAGCTGAAAGAGGACTACTTCAAGAAAATCGAGTGCTTCGACTCCGTGGAAATCTCCGGCGTGGAAGATCGGTTCAACGCCTCCCTGGGCACATACCACGATCTGCTGAAAATTATCAAGGACAAGGACTTCCTGGACAATGAGGAAAACGAGGACATTCTGGAAGATATCGTGCTGACCCTGACACTGTTTGAGGACAGAGAGATGATCGAGGAACGGCTGAAAACCTATGCCCACCTGTTCGACGACAAAGTGATGAAGCAGCTGAAGCGGCGGAGATACACCGGCTGGGGCAGGCTGAGCCGGAAGCTGATCAACGGCATCCGGGACAAGCAGTCCGGCAAGACAATCCTGGATTTCCTGAAGTCCGACGGCTTCGCCAACAGAAACTTCATGCAGCTGATCCACGACGACAGCCTGACCTTTAAAGAGGACATCCAGAAAGCCCAGGTGTCCGGCCAGGGCGATAGCCTGCACGAGCACATTGCCAATCTGGCCGGCAGCCCCGCCATTAAGAAGGGCATCCTGCAGACAGTGAAGGTGGTGGACGAGCTCGTGAAAGTGATGGGCCGGCACAAGCCCGAGAACATCGTGATCGAAATGGCCAGAGAGAACCAGACCACCCAGAAGGGACAGAAGAACAGCCGCGAGAGAATGAAGCGGATCGAAGAGGGCATCAAAGAGCTGGGCAGCCAGATCCTGAAAGAACACCCCGTGGAAAACACCCAGCTGCAGAACGAGAAGCTGTACCTGTACTACCTGCAGAATGGGCGGGATATGTACGTGGACCAGGAACTGGACATCAACCGGCTGTCCGACTACGATGTGGACCATATCGTGCCTCAGAGCTTTCTGAAGGACGACTCCATCGACAACAAGGTGCTGACCAGAAGCGACAAGAACCGGGGCAAGAGCGACAACGTGCCCTCCGAAGAGGTCGTGAAGAAGATGAAGAACTACTGGCGGCAGCTGCTGAACGCCAAGCTGATTACCCAGAGAAAGTTCGACAATCTGACCAAGGCCGAGAGAGGCGGCCTGAGCGAACTGGATAAGGCCGGCTTCATCAAGAGACAGCTGGTGGAAACCCGGCAGATCACAAAGCACGTGGCACAGATCCTGGACTCCCGGATGAACACTAAGTACGACGAGAATGACAAGCTGATCCGGGAAGTGAAAGTGATCACCCTGAAGTCCAAGCTGGTGTCCGATTTCCGGAAGGATTTCCAGTTTTACAAAGTGCGCGAGATCAACAACTACCACCACGCCCACGACGCCTACCTGAACGCCGTCGTGGGAACCGCCCTGATCAAAAAGTACCCTAAGCTGGAAAGCGAGTTCGTGTACGGCGACTACAAGGTGTACGACGTGCGGAAGATGATCGCCAAGAGCGAGCAGGAAATCGGCAAGGCTACCGCCAAGTACTTCTTCTACAGCAACATCATGAACTTTTTCAAGACCGAGATTACCCTGGCCAACGGCGAGATCCGGAAGCGGCCTCTGATCGAGACAAACGGCGAAACCGGGGAGATCGTGTGGGATAAGGGCCGGGATTTTGCCACCGTGCGGAAAGTGCTGAGCATGCCCCAAGTGAATATCGTGAAAAAGACCGAGGTGCAGACAGGCGGCTTCAGCAAAGAGTCTATCCTGCCCAAGAGGAACAGCGATAAGCTGATCGCCAGAAAGAAGGACTGGGACCCTAAGAAGTACGGCGGCTTCGACAGCCCCACCGTGGCCTATTCTGTGCTGGTGGTGGCCAAAGTGGAAAAGGGCAAGTCCAAGAAACTGAAGAGTGTGAAAGAGCTGCTGGGGATCACCATCATGGAAAGAAGCAGCTTCGAGAAGAATCCCATCGACTTTCTGGAAGCCAAGGGCTACAAAGAAGTGAAAAAGGACCTGATCATCAAGCTGCCTAAGTACTCCCTGTTCGAGCTGGAAAACGGCCGGAAGAGAATGCTGGCCTCTGCCGGCGAACTGCAGAAGGGAAACGAACTGGCCCTGCCCTCCAAATATGTGAACTTCCTGTACCTGGCCAGCCACTATGAGAAGCTGAAGGGCTCCCCCGAGGATAATGAGCAGAAACAGCTGTTTGTGGAACAGCACAAGCACTACCTGGACGAGATCATCGAGCAGATCAGCGAGTTCTCCAAGAGAGTGATCCTGGCCGACGCTAATCTGGACAAAGTGCTGTCCGCCTACAACAAGCACCGGGATAAGCCCATCAGAGAGCAGGCCGAGAATATCATCCACCTGTTTACCCTGACCAATCTGGGAGCCCCTGCCGCCTTCAAGTACTTTGACACCACCATCGACCGGAAGAGGTACACCAGCACCAAAGAGGTGCTGGACGCCACCCTGATCCACCAGAGCATCACCGGCCTGTACGAGACACGGATCGACCTGTCTCAGCTGGGAGGCGACAAAAGGCCGGCGGCCACGAAAAAGGCCGGCCAGGCAAAAAAGAAAAAGTAA
