# Supplementary figures and images for: Are the current gRNA ranking prediction algorithms useful for genome editing in plants?
Source: PLoS One. 2020 Jan 24;15(1):e0227994. doi: 10.1371/journal.pone.0227994 (PMC6980586; doi:10.1371/journal.pone.0227994)

Fig 1C. Raw gel image

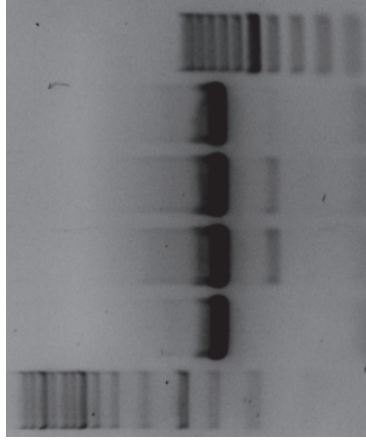

Fig 2B. Raw gel image

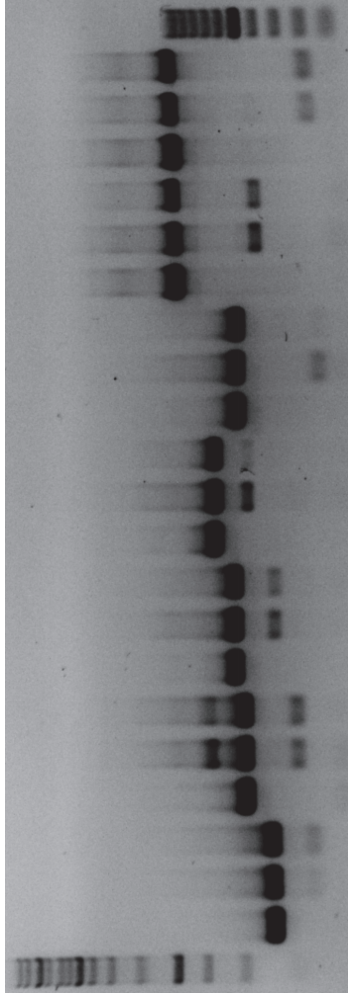

Supplement: S1 Raw images — (PDF) [file pone.0227994.s002.pdf]
